# A Novel 3D Skin Explant Model to Study Anaerobic Bacterial Infection

**DOI:** 10.3389/fcimb.2017.00404

**Published:** 2017-09-14

**Authors:** Grazieli Maboni, Rebecca Davenport, Kate Sessford, Kerstin Baiker, Tim K. Jensen, Adam M. Blanchard, Sean Wattegedera, Gary Entrican, Sabine Tötemeyer

**Affiliations:** ^1^School of Veterinary Medicine and Science, University of Nottingham Nottingham, United Kingdom; ^2^National Veterinary Institute, Technical University of Denmark Copenhagen, Denmark; ^3^Moredun Research Institute Penicuik, United Kingdom

**Keywords:** skin culture, *ex vivo* model, footrot, bacterial infection, *Dichelobacter nodosus*, ovine pro-inflammatory cytokines

## Abstract

Skin infection studies are often limited by financial and ethical constraints, and alternatives, such as monolayer cell culture, do not reflect many cellular processes limiting their application. For a more functional replacement, 3D skin culture models offer many advantages such as the maintenance of the tissue structure and the cell types present in the host environment. A 3D skin culture model can be set up using tissues acquired from surgical procedures or post slaughter, making it a cost effective and attractive alternative to animal experimentation. The majority of 3D culture models have been established for aerobic pathogens, but currently there are no models for anaerobic skin infections. Footrot is an anaerobic bacterial infection which affects the ovine interdigital skin causing a substantial animal welfare and financial impact worldwide. *Dichelobacter nodosus* is a Gram-negative anaerobic bacterium and the causative agent of footrot. The mechanism of infection and host immune response to *D. nodosus* is poorly understood. Here we present a novel 3D skin *ex vivo* model to study anaerobic bacterial infections using ovine skin explants infected with *D. nodosus*. Our results demonstrate that *D. nodosus* can invade the skin explant, and that altered expression of key inflammatory markers could be quantified in the culture media. The viability of explants was assessed by tissue integrity (histopathological features) and cell death (DNA fragmentation) over 76 h showing the model was stable for 28 h. *D. nodosus* was quantified in all infected skin explants by qPCR and the bacterium was visualized invading the epidermis by Fluorescent *in situ* Hybridization. Measurement of pro-inflammatory cytokines/chemokines in the culture media revealed that the explants released IL1β in response to bacteria. In contrast, levels of CXCL8 production were no different to mock-infected explants. The 3D skin model realistically simulates the interdigital skin and has demonstrated that *D. nodosus* invades the skin and triggered an early cellular inflammatory response to this bacterium. This novel model is the first of its kind for investigating an anaerobic bacterial infection.

## Introduction

Skin infection studies are often limited by cost prohibitive experiments and ethical concerns, leading to a dependence on *in vitro* models utilizing traditional monolayer cell culture. The limitations of monolayer cell culture have been recently recognized as they do not reflect the *in vivo* cellular processes, due to the lack of multicellular interaction (Ren et al., 2006; MacNeil, 2007; Edmondson et al., 2014). Single cell models consequently may differ in gene and protein expression from *in vivo* models (Gurski et al., 2010; Price et al., 2012). Therefore, alternative *in vitro* methods are needed to provide physiologically relevant conditions in a system that can realistically simulate mechanisms of skin infections. Three-dimensional (3D) organ culture can be used to investigate bacterial infections based on *in vitro* culture of skin explants. These tissues can be acquired from surgical procedures or post slaughter (Sanders et al., 2002; Smijs et al., 2007; Steinstraesser et al., 2010; Sidgwick et al., 2016; Wang et al., 2016). Since the multicellular interaction and tissue cytoarchitecture are preserved, 3D models are considered to be phenotypically and histologically similar to the organs and tissues *in vivo* and their importance in the development of relevant models have been widely recognized (Ren et al., 2006; Edmondson et al., 2014). Research on bacterial infections using 3D skin culture has been mainly developed for facultative anaerobic and aerobic bacteria such as *Staphylococcus aureus, Pseudomonas aeruginosa*, and *Acinetobacter* spp. (Steinstraesser et al., 2010; de Breij et al., 2012; Popov et al., 2014). In contrast, there is no established 3D skin model to study anaerobic bacterial infections.

*Dichelobacter nodosus* is an anaerobic Gram-negative bacterium and the causative agent of footrot, an interdigital skin infection of sheep (Beveridge, 1941; Egerton et al., 1969; Kennan et al., 2010). The disease is characterized by the separation of the hoof from the underlying soft tissues and causes a substantial animal welfare issue leading to a significant financial impact for farmers worldwide (Hickford et al., 2005; Wassink et al., 2010; Rather et al., 2011). The etiology is complex and virulent strains of *D. nodosus* are important in the initiation of the disease (Beveridge, 1941; Kennan et al., 2010). Virulent and benign *D. nodosus* strains differ in their ability to degrade the extracellular matrix of host tissues using extracellular proteases, whereby the virulent AprV2 protease differs from its benign counterpart AprB2 by a single amino acid change (Tyr92Arg) (Riffkin et al., 1995; Kennan et al., 2010). A recent whole genome analysis of 103 *D. nodosus* strains, isolated from eight different countries, identified that *D. nodosus* has a global conserved bimodal population with two distinct clades. Clade I was generally more associated with virulent *D. nodosus* and Clade II with benign *D. nodosus* strains (Kennan et al., 2014). It is important to highlight that these clades do not always correlate with severity of clinical presentations, as virulent *D. nodosus* can be identified in sheep without any clinical signs (Stäuble et al., 2014) and benign *D. nodosus* strains have been isolated from underrunning footrot lesions of Swedish sheep (Frosth et al., 2015). The severity of footrot is thought to be exacerbated by the intense inflammatory response to the infection. However, little is known about the host immune response to this disease. IL1β was suggested to be involved in the inflammatory response to *D. nodosus* with high levels of expression, significantly associated with abundance in naturally infected skin biopsies (Davenport et al., 2014; Maboni et al., 2017). Conversely, high expression of other cytokines/chemokines, such as CXCL8, IL6 and IL17, were not correlated with *D. nodosus* abundance (Maboni et al., 2017). Additional evidence of the role played by IL1β was obtained using a single cell type model, where increased expression was measured in ovine fibroblasts stimulated with *D. nodosus* (Davenport et al., 2014). To date, this monolayer fibroblast model is the only *in vitro* cell-based method available to investigate footrot. In this context, we hypothesized that a 3D skin model could more realistically simulate the ovine interdigital skin microenvironment and *D. nodosus* could penetrate the skin, triggering an early local cellular inflammatory response.

In this study we describe the development of a novel 3D *ex vivo* skin culture model to study the infection caused by *D. nodosus* using ovine interdigital skin explants. We demonstrate that *D. nodosus* can invade the skin and alter the expression of key inflammatory markers. Tissue and cell viability was assessed and the early cellular response to bacteria was investigated by quantification of IL1β and CXCL8 protein release in the medium. In summary, this method is simple, robust and can be applied to investigate other anaerobic bacterial infections.

## Materials and methods

### Sample collection

Healthy ovine feet were collected from sheep at a local abattoir post-slaughter. Feet with good hoof conformation and with apparently healthy interdigital skin were selected. Environmental contaminants were removed from the foot and interdigital space using 70% ethanol and antimicrobial skin cleanser (Hibiscrub®) before removal of the hair using scissors. The entire ovine interdigital skin was removed using a sterile scalpel. Skin explants were immediately immersed in transport media after collection [DMEM-HAM'S F12 1:1 (Sigma Aldrich®), Penicillin+Streptomycin 0.01 μg/ml (Gibco®), Amphotericin B 0.01 μg/ml (Lonza®), Gentamicin 5 μg/ml (Sigma Aldrich®), L-glutamine 0.01 μg/ml (Gibco®)]. Biopsies were collected aseptically from each interdigital skin fragment using an 8 mm punch biopsy (National Veterinary Service, UK). The surface of the biopsy was gently incised 4 times with a scalpel blade to simulate naturally occurring damage to the skin (required for the invasion of *D. nodosus*).

### Assembling the 3D skin explant model

Biopsies were immersed in wash medium [DMEM-HAM'S F12 1:1, Penicillin+Streptomycin 0.01 μg/ml (Gibco®), Amphotericin B 0.01 μg/ml (Lonza®), Gentamicin 2 μg/mL (Sigma Aldrich®)], pre-heated to 37°C, and incubated at room temperature for 15 min. The media change and incubation steps were repeated 3 times.

**Figure 1:**
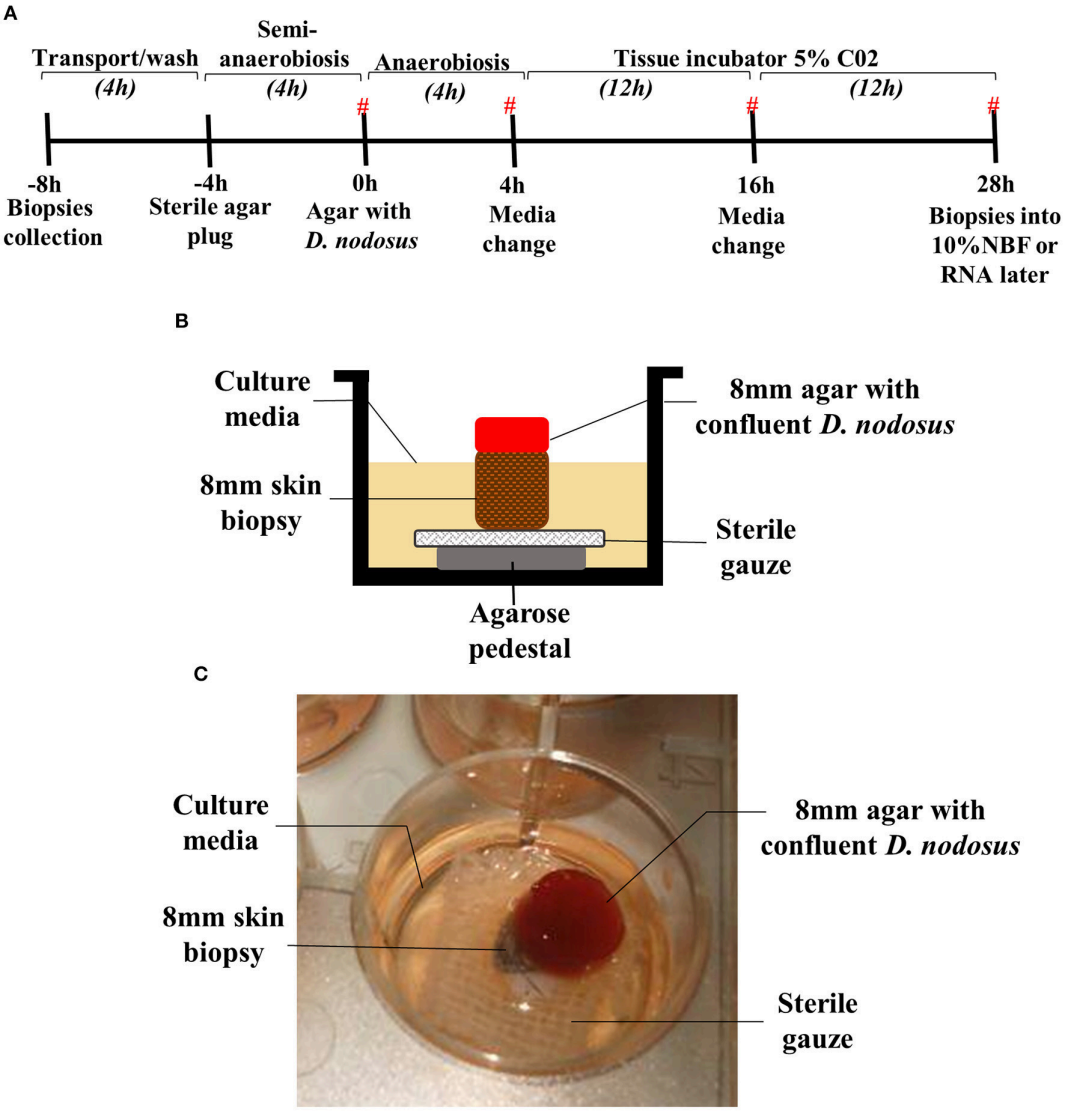
Assembled 3D skin explant model for anaerobic bacterial infection. **(A)** Timeline of the model infection highlighting the time points where biopsies were placed into RNAlater or 10% NBF. (#) Time points where the culture media were collected for cytokine measurement. Culture media without antibiotics was used between −4 and 4 h. Culture media containing antibiotics was used between 4 and 28 h after bacterial exposure. **(B)** Schematic overview of the assembled model at 0–28 h (exposure to *Dichelobacter nodosus*). **(C)** Photo of the assembled model in a 12 well-plate.

To provide a physical support for the biopsies, an agarose pedestal (500 μl of 1.2% w/v agarose (Thermo Scientific®) in DMEM-HAM'S F12 1:1 (Sigma-Aldrich®) covered with 0.5 cm^2^ sterile surgical gauze was assembled in a 12 well-plate (Thermo Scientific®). The skin biopsy was placed on the top of the surgical gauze and agarose pedestal (Figure 1B).

For the initial assessment of model viability, five time points were evaluated for up to 76 h of mock infection: biopsy collection −8 h, mock infection 0, 28, 52, and 76 h post mock infection. Two biopsies were collected at each time point; one placed into RNAlater (Sigma-Aldrich®) for DNA extraction and the other one was fixed in 10% v/v Neutral Buffer Formalin (NBF) for histological investigation. The model viability was analyzed assessing at each time point the tissue integrity and architecture (scoring of 2 H&E stained sections), and cell viability by TUNEL stain (5 non-overlapping images for each, epidermis and dermis).

### Skin explants infection with *D. nodosus*

Four experiments using explants from different sheep collected in different occasions were performed. A microenvironment with restricted oxygen supply was produced by placing a sterile agarose plug (300 μl of 0.8% w/v agarose in DMEM-HAM'S F12 1:1) on the top of the biopsy, which was placed on the top of the agarose pedestal and surgical gauze, and incubated in a humidified tissue incubator at 37°C (Heracell 150i, Thermo Fisher Scientific®) with 5% CO_2_ for 4 h. 1 mL of culture media without antibiotics [DMEM high glucose (Gibco®) + DMEM HAM'S F12 1:1 (Sigma Aldrich®) in a proportion 3:1, Amphotericin B 0.25 μg/ml (Lonza®), L-glutamine 0.01 μg/ml (Gibco®), 10% Fetal Bovine Serum (Gibco®)] was placed in each well. The infection of the explants with *D. nodosus* was performed using an 8 mm punch biopsy to aseptically collect a Fastidious Anaerobic Agar plug confluent with *D. nodosus* strain MM261 (*aprV2*, Clade I, which correlates with virulent strains) or strain MM277 (*aprB2*, Clade II, which correlates with benign strains). The agar plug with *D. nodosus* was placed on the top of the biopsy and 1 mL of culture media without antibiotics was added in each well. The plate was incubated anaerobically (Oxoid AnaeroGen, Thermo Scientific®) at 37°C for 4 h. Post 4 h, maintenance of the model included culture media containing antibiotics [DMEM High Glucose (Gibco®) + DMEM HAM'S F12 1:1 (Sigma Aldrich®) in a proportion 3:1, Penicillin+Streptomycin 0.01 μg/ml (Gibco®), Gentamicin 5 μg/ml (Sigma Aldrich®), Amphotericin B 0.25 μg/ml (Lonza®), L-glutamine 0.01 μg/ml (Gibco®), 10% v/v Fetal Bovine Serum (Gibco®)] in a humidified tissue incubator with 5% C0_2_for 24 h. The media were changed 4 h post infection and then every 12 h keeping the same agar plug with *D. nodosus* placed on the top of the biopsy. Culture media from all time points were collected and stored at −80°C for further cytokine measurement. After 28 h of infection with *D. nodosus*, biopsies were placed into RNAlater for further DNA extraction or 10% v/v NBF for histology. For DNA extraction each tissue was cut into approximately 4 × 4 mm pieces and incubated with 180 μl of tissue lysis buffer (ATL) with the addition of 20 μl of proteinase K (20 mg/ml) (Qiagen) at 56°C for 3 h. DNA was isolated using the QIAamp cador kit according to manufacturer's recommendations and eluted in 50 μl AVE buffer (Qiagen). The DNA was used for *D. nodosus* quantification by qPCR, targeting the 16S rRNA gene (Forward primer: 5′-CGGGGTTATGTAGCTTGCTATG-3′, Reverse primer: 5′-TACGTTGTCCCCCACCATAA-3′, probe: 5′FAM-TGGCGGACGGGTGAGTAATATATAGGAATC-TAMRA-3′) (Frosth et al., 2012). qPCR data were normalized to pg of *D. nodosus* DNA present in the total DNA extracted from each biopsy. The cut off of 0.1pg was assigned for negative qPCR results. The *D. nodosus* load present on an 8 mm agar area was estimated using the total DNA extracted from *D. nodosus* confluent on the 8 mm surface and confirmed by qPCR.

### Skin tissue fixing, processing, and staining

Interdigital skin biopsies were fixed for 48 h at 4°C using an extended tissue processing protocol (60 min in dH_2_O, 4 h in 50% ethanol, 4 h in 70% ethanol, 16 h in 90% ethanol, 4 h in 100% ethanol, 4 h in xylene, 2 h of wax immersion at 60°C). Paraffin wax embedded tissues were soaked in 10% (v/v) ammoniated water while 3 and 6 μm thick sections were cut from each block by microtome (Leica RM2255®) and placed on polystyrene microscope slides (Thermo Scientific®). H&E stain (Sigma-Aldrich®) was used to allow visual assessment of the 6 μm tissue sections (see Supplementary Table 1 for H&E protocol). All slides were mounted with Distyrene Plasticizer Xylene (DPX) (Sigma-Aldrich®) and were analyzed by microscopy (Leica DM 2500®). A board certified veterinary pathologist, blinded to slide identity, evaluated 54 H&E-stained biopsy sections to assess tissue integrity and architecture. A qualitative, semi-quantitative scoring system was developed to grade the tissue integrity and architecture according to histopathological features (Table 1 and Supplementary Figure 2).

**Table 1:** Qualitative, semi-quantitative scoring system used to evaluate the tissue integrity and viability of the ovine skin explants from the 3D culture model.

**Score**	**Histopathology (changes observed)**	**Tissue viability**
0 (plus signs stated below)	- Acute ischaemic degeneration of epidermal cells (necrosis) - Severe extent of all epidermal and dermal changes described below	Tissue is not viable
1 Marked (plus signs stated below)	- Multifocal subepidermal clefting - Basal cell vacuolisation (epidermal degeneration) - Nuclear pyknosis and detachment of eccrine glands, leukocytes and epidermal cells showing signs of autolysis - Multifocal pyknosis of basal cells with degenerating neutrophils	Tissue is viable
1.5 Moderate to marked (plus signs stated below)	- Apoptotic keratinocytes - Vacuolation of basal cells - Supepidermal clefting - Eccrine glands and peripheral nerves show signs of autolysis, as do some follicular epithelial cells (detached)	Tissue is viable
2 Moderate (plus signs stated below)	- Prominent pyknotic nuclei in stratum spinosum - Some subasilar clefting around follicles - Few sloughed epithelial cells in glands	Tissue is viable
2.5 Mild to moderate (plus signs stated below)	- Scattered apoptotic keratinocytes	Tissue is viable
3 Mild	- Normal tissue architecture and viability and occasionally few sloughed epithelial cells within eccrine glands	Tissue is viable

The modified DeadEnd Colorimetric TUNEL System (Promega®) was used to detect DNA fragmentation. Assays were performed according to the manufacturer's instructions using 6 μm tissue sections. Cells were stripped of proteins and made permeable by incubation with 20 μg/ml of proteinase K solution for 20 min and DAB 20x chromogen solution incubation was performed for 3 min. The sections were counterstained with haematoxylin solution for 5 s, rinsed in tap and deionized water. All slides were mounted with DPX. Negative controls were obtained by omitting the TdT enzyme from the TdT mix in the reaction (according to manufacturer's instructions). Positive controls were generated by treating a skin section with DNAse (Promega®). In order to assess the number of dead and live cells, 5 non-overlapping images from each, the epidermis and the dermis of each biopsy tissue section, were used (200x total magnification). Images were captured and analyzed by microscopy (CTR500 microscope, Leica Microsystems®). All cells from all images (epidermis and dermis) from each sample were counted using Fiji/ImageJ-win64 software (https://fiji.sc/, October 2016) and the percentage of dead and live cells were calculated for all 10 images/sample based on the total number of dead and live cells obtained. Brown stained nuclei were assessed as dead cells, whereas blue stained nuclei were assessed as live cells due to counterstain with haematoxylin.

### Fluorescent *in Situ* hybridization (FISH) for *D. nodosus* and eubacteria detection and localization

FISH analysis was performed as previously described (Rasmussen et al., 2012). The 16S rRNA-targeting oligonucleotide probes for *D. nodosus* (S-S-D.nodosus-443 5′-CATGCACCGTTCTTCACT-3') and eubacteria domain (S-D-eub-338-alexa 5′-GTCATTCCATCGAAACATA-3′) labeled with fluorescein isothiocyanate (FITC) or Cy3 were used on 3 μm tissue sections. Hybridization was carried out at 46°C with hybridization buffer (100 mM Tris, pH 7.2, 0.9 M NaCl, 0.1% sodium dodecyl sulfate) containing 5 ng/ml of each applied oligonucleotide probe (usually double hybridizations with a FITC and CY3 labeled probe) for 16 h in a Sequenza slide rack (Thermo Shandon, Cheshire, United Kingdom). Slides were then washed with prewarmed (46°C) hybridisation buffer for 3 × 3 min followed by wash with prewarmed (46°C) washing buffer (100 mM Tris, pH 7.2, 0.9 M NaCl) for 3 × 3 min. Finally they were rinsed in water, air dried and mounted with Vectashield (Vector Laboratories Inc.®) for epifluorescense microscopy using an Axioimager M1 epifluorescense microscope. Images were obtained using an AxioCAM MRm version 3 FireWiremonochrome camera (Carl Zeiss®).

### Cytokine/chemokine measurement by enzyme-linked immunosorbent assay (ELISA)

IL1β and CXCL8 were measured in the tissue culture media collected from −4 to 0 h, 0 to 4 h, 4 to 16 h, and 16 to 28 h post infection, using specific sandwich ELISAs as previously described (Doull et al., 2015). Recombinant ovine IL1β (Kingfisher®) and recombinant ovine CXCL8 (Doull et al., 2015) were used as quantifiable ELISA standards. Cytokine concentrations were calculated using a polynomial quadratic regression in GraphPad Prism version 7b.

### Statistical analysis

One-way analysis of variance followed by Dunn's multiple comparisons test was applied for *D. nodosus* load as well as for percentage of TUNEL positive cells. Mann Whitney (non-parametric test) was used for IL1β release between infected and mock-infected biopsies across the time course. Mean, media, standard deviation (SD) and all other analysis were performed using GraphPad Prism version 7b. A *P* ≥ 0.05 was considered significant.

## Results

### Assembling the 3D skin model

The assembled 3D skin infection model consisted of an agarose pedestal overlaid with surgical gauze to provide support for the biopsy in the well of a 12 well-plate (Figure 1B). The skin biopsy was then placed upon the surgical gauze. The skin explants were initially maintained during 4 h under atmospheric oxygen, but the biopsy surface area was sealed with a sterile agar plug to simulate the natural ovine feet microenvironment with a moist surface and restricted oxygen supply (Figures 1A,B). For the infection experiment, the sterile agar plug was replaced by an agar plug with confluent *D. nodosus* placed on the top of the biopsy and incubated under anaerobiosis for 4 h, followed by 24 h of aerobic incubation to ensure skin tissue survival (Figure 1A). For the mock-infection viability experiment, the sterile agar plug was replaced by another sterile agar plug and the model was maintained under the same temperature and incubation conditions as described above. Up to 1 ml of medium was added up to the top level of the explant to avoid flooding of the explant or removal of the agar plug.

### Tissue integrity and cell viability maintenance during 76 h of model incubation

A 76 h time course mock-infection experiment was performed to identify optimal conditions for oxygen dependent tissue to remain viable, while also allowing transient anaerobic incubation essential for the pathogen (Supplementary Figure 1A). The viability of explants was assessed by tissue integrity and cell death at five time points: immediately after sample collection −8 h, mock-infection 0, 28 h, 52 and 76 h post mock infection. Using the scoring system developed to grade the tissue integrity and architecture according to histopathological features (Table 1 and Supplementary Figure 2), it was visualized that skin explants maintained the normal tissue architecture for up to 28 h of incubation with early signs of tissue degeneration in biopsies incubated for more than 52 h (Supplementary Figure 1B). The early signs of tissue degeneration included basal cell vacuolisation, subepidermal clefting, sloughed cells in glands and follicular epithelial cells (Supplementary Figure 1B). Additionally, cell viability was investigated through the quantification of DNA fragmentation using the TUNEL stain. The percentage of live cells in the epidermis decreased from −8 h [99% (2497/2524)) to 76 h of incubation (45% (296/660)] (Supplementary Figures 1C, 3). A high percentage of live cells was found in the dermis in all time points, with a slight decrease from −8 h [99.3% (2092/2105)] to 76 h [84% (952/1131)] (Supplementary Figure 1C). As expected, biopsies fixed at the abattoir post slaughter had a low level of dead cells. The cell viability data confirmed the tissue integrity data as skin explants presented a high percentage of live cells (TUNEL) and well preserved tissue architecture (H&E tissue integrity scores) in both epidermis and dermis (Supplementary Figure 1C). As the 28 h incubation showed more than 50% of the epidermal and dermal cells were alive and the tissue architecture was viable (score 1.5) (Supplementary Figure 1C), this time point was used as the implemented cut-off for further infection experiments.

### Ovine skin explant as an infection model for anaerobic bacteria

**Figure 2:**
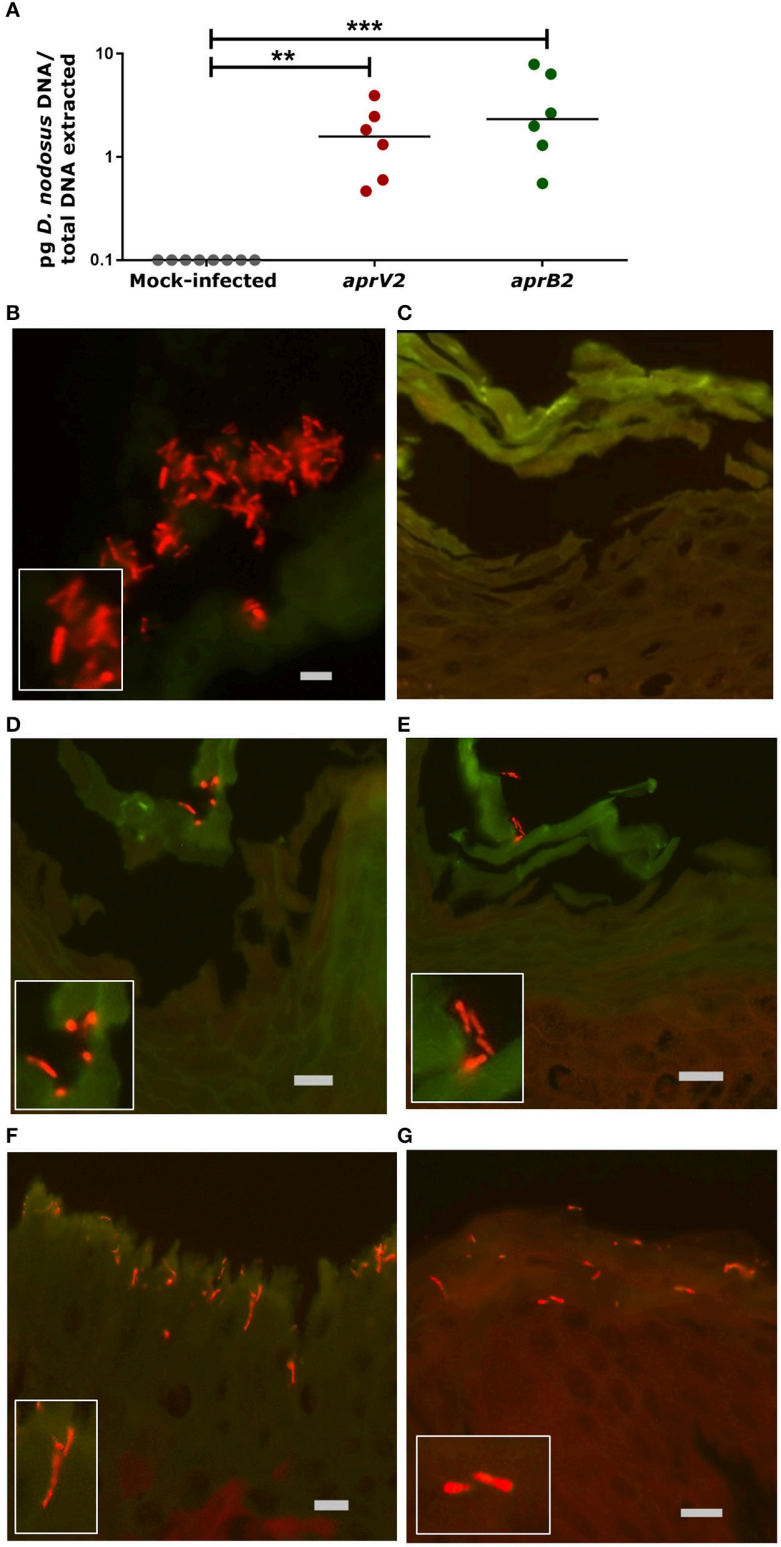
Infection of the 3D skin explant model with *Dichelobacter nodosus*. **(A)** Detection of *D. nodosus aprV2* and *aprB2* strains in the skin explants using quantitative PCR after 28 h of infection. Each point indicates a single biopsy. 0.1 = results below of the limit of detection. Mean is represented by black bars. Data were analyzed by Dunn's multiple comparisons test using GraphPad Prism ^**^P ≤ 0.01, ^***^P ≤ 0.001. **(B–G)** Fluorescent *in situ* Hybridization on ovine interdigital skin explants after 28 h of infection with *D. nodosus*. **(B)** Positive tissue control with *D. nodosus* reference strain (CCUG 27824) (red/orange); **(C)** Uninfected negative control; **(D,E)** Demonstration of *aprB2* and **(F,G)** *aprV2 D. nodosus* (red/orange) on the surface or migrating within the epidermal layers. **(C–E)** were hybridized with the Cy3 labeled *D. nodosus* probe only, while **(F,G)** were hybridized with both, the *D nodosus* and the eubacteria probe. Squares located on the bottom/left side show the zoomed image. Scale bars (gray): **(B)**, 5 μm, **(C–G)**, 10 μm.

The *D. nodosus* of the 8 mm agar plug was quantified by qPCR to estimate the copy number used to infect each biopsy (1.13 × 10^4^ of *aprV2 D. nodosus* DNA, strain MM261, and 7.16 × 10^4^ of *aprB2 D. nodosus* DNA, strain MM277). The presence of *D. nodosus* DNA was confirmed in all infected biopsies with *aprV2* (2.13 × 10^4^ *D. nodosus*/biopsy, *n* = 6) and *aprB2 D. nodosus* (1.11 × 10^5^ *D. nodosus*/biopsy, *n* = 6) using qPCR. In contrast, all mock-infected biopsies (*n* = 8) were negative for *D. nodosus* (Figure 2A).

Fluorescent *in situ* hybridization (FISH) was used to localize *D. nodosus* within the skin explant. *D. nodosus* bacterial cells were identified in the positive porcine lung tissue control (Figure 2B) and in all biopsies infected with either *aprV2* (*n* = 4) or *aprB2 D. nodosus* (*n* = 4; Figures 2D–G). Negative tissue control (*n* = 1; Figure 2C) and all mock-infected skin control biopsies (*n* = 5) were negative for *D. nodosus* (Table 2). *D. nodosus* was identified on the skin surface (Figures 2D–E) and also invading the superficial layers of the epidermis (Figures 2F,G). *D. nodosus* cells were visualized throughout these layers suggesting that the bacteria were moving away from the initial skin laceration site. *D. nodosus* was not identified in the dermis. A general eubacterial domain probe did not detect the presence of other bacteria in any infected or mock-infected explants. This showed that both *aprV2* and *aprB2* strains of *D. nodosus* have the ability to migrate into the damaged skin layers during 28 h of incubation.

**Table 2:** Fluorescent *in situ* Hybridization of targeted *Dichelobacter nodosus* on ovine interdigital skin tissue samples from the 3D skin culture model.

**Samples**	**FISH score system median (min–max)**
Mock-infected controls (*n* = 5)	0 (0)
Infected with *aprV2 D. nodosus* (*n* = 4)	1.5 (1–2)
Infected with *aprB2 D. nodosus* (*n* = 4)	2.5 (1–3)

*Score system: 0, no invasive bacteria; 1, low number of invasive bacteria from 1 to 20; 2, moderate number of invasive bacteria from 20 to 200; 3, high number of invasive bacteria from 200 to uncountable bacterial cells (Modified from Rasmussen et al., 2012). Min, minimum; max, maximum*.

### Bacterial infection had minimal impact on skin explants viability over 28 h

**Figure 3:**
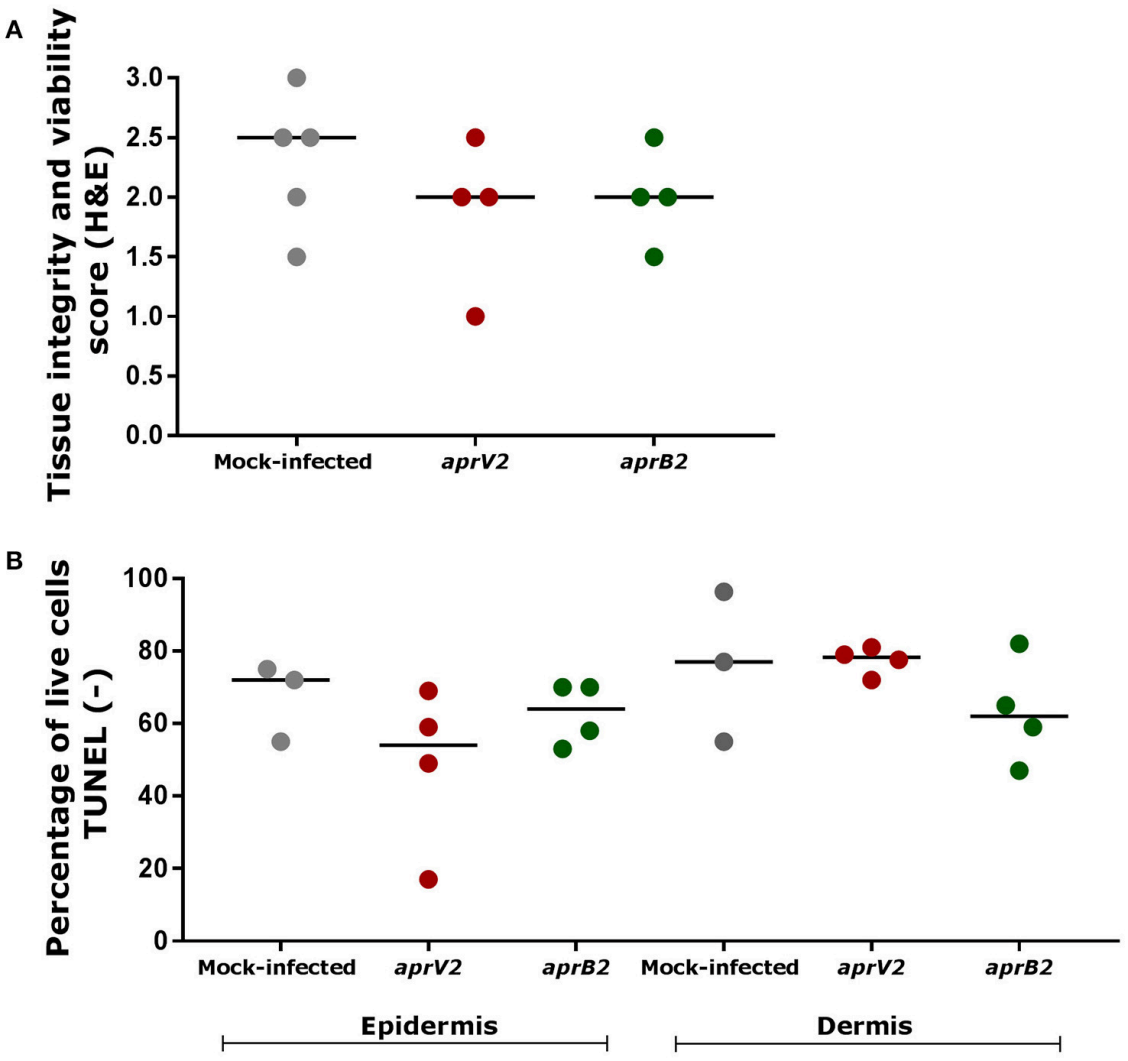
Tissue integrity and cell viability assessment of skin explants cultured in the 3D model after 28 h of infection with *aprV2* and *aprB2 Dichelobacter nodosus*. **(A)** Tissue integrity and viability score (H&E) after 28 h of bacterial infection for both epidermis and dermis. Histological score 0: tissue is not viable; score 1: viable tissue, but showing marked signs of tissue degeneration; score 1.5: viable tissue, but showing moderate to marked signs of tissue degeneration; score 2: viable tissue, but showing moderate signs of tissue degeneration; score 2.5: viable tissue, but showing mild to moderate signs of tissue degeneration; score 3: tissue is viable and shows only few mild signs of tissue degeneration. **(B)** Percentage of live cells (TUNEL negative, no DNA fragmentation) in the epidermis and dermis of skin explants after 28 h of bacterial infection. Each point indicates a single explant. Black bars indicate median. TUNEL data were analyzed by Dunn's multiple comparisons test using GraphPad Prism.

The tissue integrity and cell viability from infected and mock-infected skin explants were assessed to investigate the histological effects of bacterial infection on the tissue viability and maintenance. *D. nodosus* infection had little impact on tissue integrity/architecture or on cell viability after 28 h (Figure 3). Tissue viability of all mock-infected control biopsies was maintained over 28 h of incubation (median histopathology score 2.5), with the exception of one biopsy (score 1.5) (Figure 3A; See Table 1 for description of histopathology scores). Similarly, infected biopsies maintained the tissue integrity (median histopathology score 2), with the exception of two biopsies (scores 1 and 1.5) (Figure 3A). All mock-infected controls and infected explants presented more than 50% (69–98.9%) of epidermal and dermal cells alive (TUNEL stain) after 28 h of bacterial exposure, with the exception of one biopsy infected with the *aprV2 D. nodosus* strain (Figure 3B). There was no statistically significant difference between infected and mock-infected skin explants for the percentage of live cells.

### Pro-inflammatory cytokine/chemokine released in the culture media

**Figure 4:**
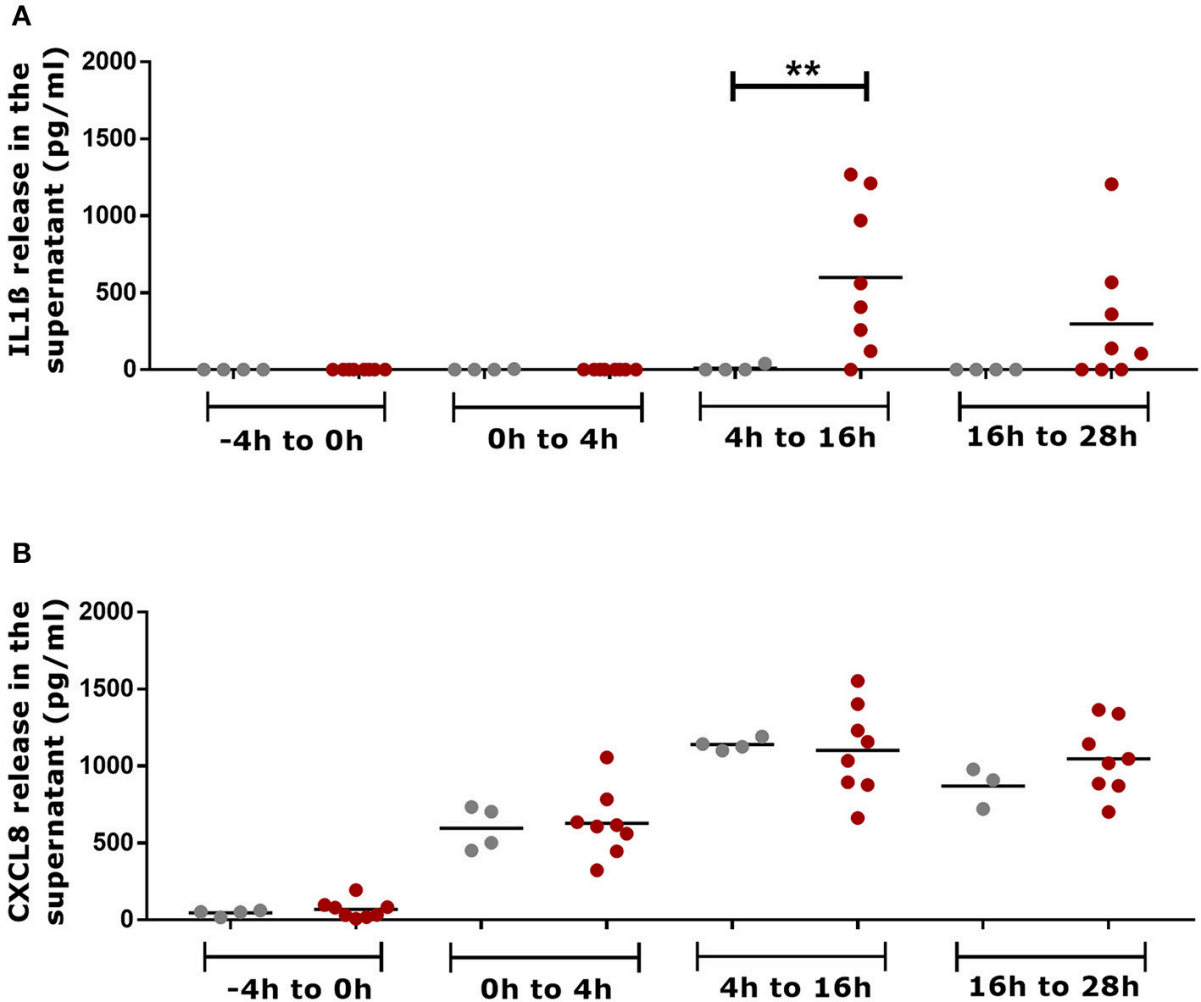
Measurement of pro-inflammatory mediators in the culture media supernatants of the 3D skin explant model using IL1β and CXCL8 specific ELISAs. **(A)** Accumulative detection of IL1β in mock-infected and infected explants with *aprV2* and *aprB2 Dichelobacter nodosus* at different time points. **(B)** Accumulative detection of CXCL8 in mock-infected and infected explants. −4–0 h = total accumulation in the media supernatant during 4 h of semi-anaerobic conditions (mock infection); 0–4 h = total accumulation in the media supernatant 4 h post infection with *D. nodosus*; 4–16 h total accumulation in the media 4–16 h post infection with *D. nodosus* (12 h) and 16–28 h = total accumulation in the media 16–28 h post infection with *D. nodosus* (12 h). Mann Whitney test (non-parametric) was performed for comparison between mock-infected and infected explants using GraphPad Prism ^**^P ≤ 0.01. Each point indicates a single explant. Gray points indicate mock-infected and red points indicate *D. nodosus* infected explants. Black bars represent mean.

Skin explants infected with *aprV2* and *aprB2 D. nodosus* released IL1β in the culture media, which accumulated between 4–16 h and 16–28 h post infection (Figure 4A). At 4–16 h, the concentration of IL1β in infected biopsies (600 ± 492 IL1β pg/mL, *n* = 8) was statistically significantly greater than in mock-infected controls (10 ± 20 IL1β pg/mL, *n* = 4) (*P* ≤ 0.01). All media samples from mock-infected and infected biopsies collected at −4 h mock infection and 4 h after infection (4 h of accumulation) were negative or showed very low concentrations of IL1β (Figure 4A). Mock-infected controls and infected skin explants released similar concentrations of CXCL8 in the culture media that were accumulated between 0–4 h, 4–16 h and 16–28h post infection (Figure 4B). CXCL8 was not released in the culture media between −4 and 0 h of mock infection time (Figure 4B).

## Discussion

This study presents a novel 3D skin model culture using *ex vivo* explants for infection with anaerobic bacteria. The key findings include that both, *aprV2* and *aprB2* strains of *D. nodosus*, were able to migrate from the agar plug into tissues after 28 h of exposure, and those tissues responded to bacterial infection with the release of IL1β into the culture supernatant. To our knowledge, this is the first study to establish a 3D culture model using ovine skin explants to investigate an anaerobic bacterial infection. The use of *ex vivo* ovine skin in 3D culture has the advantage that explants are easily available from abattoirs, where they are by-products of the slaughter process.

Migration of *D. nodosus* into the skin explants was confirmed by qPCR and FISH. *D. nodosus* was clearly visualized, not only on the skin surface, but also invading the stratum corneum and superficially in the stratum granulosum in all infected biopsies. These findings correspond with an early report that *D. nodosus* was restricted to the superficial epidermal layers of the ovine skin (Egerton et al., 1969), and those by Witcomb et al. (2015), who also localized *D. nodosus* by FISH within the superficial epidermal layer of ovine biopsies naturally affected by footrot. We further investigated the initial inflammatory response after bacterial challenge to determine the immunological functionality of the cultured skin. Importantly, all mock-infected controls did not release IL1β suggesting this cytokine was primarily released in response to *D. nodosus* infection. IL1β has a range of stimulatory effects on immune cells playing a key role in the innate immunity of the skin (Arend et al., 2008). It has been reported elsewhere that the mRNA expression levels of IL1β and CXCL8 increase after human skin is exposed to *Acinetobacter* spp., while IL1β protein is undetectable in the culture media (de Breij et al., 2012). We have shown previously that IL1β mRNA is expressed in footrot samples (Davenport et al., 2014; Maboni et al., 2017). mRNA expression of *TLR4* and *TLR2* was also increased in these samples (Davenport et al., 2014), which likely resulted in the activation of signaling pathways culminating in the transcription and translation of the inactive precursors of IL1β. Processing of the IL1ß precursor and release into the medium demonstrates that the inflammasome multiprotein complex is involved in the inflammatory response triggered by *D. nodosus*. The inflammasome comprises of the activation of caspase-1 protease, which is responsible for the proteolytic processing of the IL1β precursor hence catalyzing the posttranslational mechanism that is required for the secretion of the bioactive form of this cytokine (Fantuzzi et al., 1997; Dinarello, 2006).

CXCL8 plays a role as a potent chemoattractant for neutrophils, basophils and T cells as well as having an effect on the proliferation of keratinocytes (Tuschil et al., 1992). CXCL8 in the skin is mainly produced by keratinocytes, which increases the expression of this chemokine in response to inflammatory stimuli (Cataisson et al., 2006). Here we found similar concentrations of CXCL8 obtained from mock-infected and infected explants with an increased release from 0 to 28 h. The high levels of CXCL8 observed in the mock-infected controls in response to the model incubation conditions may have masked any response to *D. nodosus*. However, we have found previously that high mRNA expression levels of *CXCL8* were not associated with high *D. nodosus* load in naturally infected skin biopsies (Maboni et al., 2017). In contrast, *CXCL8* has been reported to be significantly more expressed in response to *Staphylococcus aureus, Pseudomonas aeruginosa* and *Acinetobacter* spp. in human skin infection models (Steinstraesser et al., 2010; de Breij et al., 2012). In the context of anaerobic bacterial infections, mRNA expression of IL1α, CXCL8, TNFα and human beta defensin (hBD)-2 were stimulated in keratinocytes by *Propionibacterium acnes*, an anaerobic bacterium (Lee et al., 2010). Also, IL17 was shown to be essential in the host defense against *Staphylococcus aureus*, a facultative anaerobic bacterium (Cho et al., 2010). The skin model developed in this study could be used to investigate the release of these molecules in response to other anaerobic bacteria.

Natural occurrence of footrot requires a damp and damaged skin microenvironment to allow *D. nodosus* to establish in the interdigital skin and to initiate under-running footrot lesions (Beveridge, 1941; Kennan et al., 2010). *In vivo* infection trials simulated the natural occurrence of footrot maintaining the sheep feet under wet conditions that allowed maceration of the interdigital skin prior to bacterial infection (Beveridge, 1941; Egerton et al., 1969; Kennan et al., 2001, 2010; Knappe-Poindecker et al., 2014). In order to replicate these conditions *in vitro*, the epidermis of each explant was lacerated before infection with *D. nodosus*. Skin explants were never allowed to dry out during transport and kept hydrated with restricted oxygen supply through the use of an agar plug, throughout the experiment. In addition, footrot has been reported to be a localized foot infection with little or unknown involvement of the ovine systemic response (Bhardwaj et al., 2014; Davenport et al., 2014). This model therefore represents a suitable approach to investigate footrot in the context of local skin architecture and cellular components.

Tissue integrity and cell viability of the skin explants were maintained over 28 h of incubation, with more marked signs of tissue degeneration in biopsies incubated for more than 52 h. Aspects that may have affected tissue survivability include animal age and breed, equal distribution of nutrients within the tissue, culture media pH and antimicrobials toxicity in the tissue explants. In this study, a heterogeneous population of sheep sampled at the abattoir from different breeds, ages and underlying subclinical disease may have impacted on tissue viability, as observed in mock-infected biopsies presenting variable integrity scores and percentage of live cells (Figure 3).

We showed that FISH targeting a general eubacterial domain did not detect the presence of other bacteria in any infected or mock-infected explants, which were likely eliminated by the antimicrobial agents of the washing medium. Although antibiotics and antifungal agents were essential in the medium to prevent contamination, they may have had a detrimental effect on tissue survivability. Since 3D models lack circulatory and excretion systems, it is likely that cytotoxic effects may happen due to the accumulation of antimicrobials in the tissue explants (Levy, 2000; Gibson et al., 2008; Costa et al., 2016). Considering that *D. nodosus* and other microorganisms may have a synergistic relationship in the pathogenesis of footrot (Maboni et al., 2016, 2017), the clearance of skin commensal microorganisms may also have an impact on the mechanism of *D. nodosus* establishment in the skin explants as well as the immune response. As expected, DNA fragmentation investigated through the TUNEL stain revealed that the percentage of live cells in the epidermis decreased from −8 h (99%) to 76 h of incubation (45%). Part of the cell death detected in the skin explants might be associated with the normal life cycle of keratinocytes, whereby DNA fragmentation for apoptosis and differentiation mechanisms occur to allow cornification of the keratinocytes that establish a tight barrier of dead cells protecting the skin (Lippens et al., 2005).

The model developed in this study could be used to generate new insights into the pathogenesis of ovine footrot as well as other bacterial infections. We envision this model as an *in vitro* alternative to investigate the synergistic role of *D. nodosus* and other bacterial species involved in footrot lesions such as *F. necrophorum, Treponema* spp. and *Mycoplasma* spp. (Beveridge, 1941; Frosth et al., 2015; Maboni et al., 2017). Further studies could investigate the role of twitching motility in the early stages of infection. Cytokine arrays could be applied to investigate a wider range of inflammatory molecules triggered in response to bacterial exposure. Importantly, the skin explant model could be improved in terms of incubation period. A time longer than 28 h of model exposure to bacteria would inform whether this model is conducive to bacterial replication as well as bacterial invasion studies. Extending the viability of the explant model could be achieved by developing a perfusion system to optimize the nutrient supply or developing a set up where more oxygen can be supplied to the tissues.

In summary, a novel skin explant model was developed using ovine *ex vivo* interdigital skin biopsies. Both, *aprV2* and *aprB2 D. nodosus* migrated into the skin layers and IL1β and CXCL8 were released in the culture media indicating the tissues were alive after 28 h of bacterial exposure. IL1β in particular was shown to be released in response to *D. nodosus* challenge. We demonstrated a proof of principle that the anaerobic bacterium *D. nodosus* could invade the 3D skin explant model and that the expression of key inflammatory markers could be quantified.

## Author contributions

Conception and design of study: ST and GM. Acquisition and analysis of data: GM, RD, KS, KB, TJ, AB, SW, GE, and ST. Drafting of article and/or critical revision: GM, RD, KB, TJ, AB, SW, GE, and ST. All authors approved the final article.

### Conflict of interest statement

The authors declare that the research was conducted in the absence of any commercial or financial relationships that could be construed as a potential conflict of interest.

## Supplementary material

Supplementary Figure 1 Viability of the interdigital skin explants over a time course of 76 h (mock-infected explants). **(A)** Timeline of viability experiment where one biopsies was placed into RNAlater and one into 10% NBF at each time point. −8h (biopsy fixed at the abattoir a few minutes after sheep slaughter); 0 h (biopsy fixed at MOCK infection time); 28 h (biopsy fixed 28 h after MOCK infection time); 52 h (biopsy fixed 52 h after MOCK infection time); 76 h (biopsy fixed 76 h after MOCK infection time). **(B)** H&E images illustrating the overall maintenance of the tissue structure and viability over 76 h of incubation. Early signs of tissue degeneration were more marked at 52 and 76 h of incubation including apoptotic keratinocytes (1), basal cell vacuolisation (2), subepidermal clefting (3) (scale bars: 100 μm). **(C)** Comparison of cell viability (TUNEL) and tissue integrity (H&E scores) in the epidermis and in the dermis of ovine interdigital skin. Histological score 0: tissue is not viable; score 1: viable tissue, but showing marked signs of tissue degeneration; score 1.5: viable tissue, but showing moderate to marked signs of tissue degeneration; score 2: viable tissue, but showing moderate signs of tissue degeneration; score 2.5: viable tissue, but showing mild to moderate signs of tissue degeneration; score 3: tissue is viable and shows only few mild signs of tissue degeneration. Mean and standard deviation of live cells from 5 non-overlapping images from the epidermis are shown. For H&E analysis, 2 slides were analysed for each single biopsy; for all time points, both slides received the same histopathological score. Black horizontal line indicates histopathology score 1, the minimal score for a tissue to be deemed viable. Blue bars show proportion of TUNEL-negative cells. Pink bars indicate tissue integrity and viability score (H&E). Each bar indicates single biopsies.

Supplementary Figure 2 H&E images illustrating examples of the qualitative, semi-quantitative scoring system used to evaluate skin explants viability (mock-infected explants). **(A)** Histological score 0 (tissue not viable) 1 = subepidermal clefting, 2 = basal cell vacuolisation, 3 = necrotic area. **(B)** Histological score 1 (viable tissue), marked 1 = subepidermal clefting, 2 = basal cell vacuolisation. **(C)** Histological score 2 (viable tissue), mild to moderate, 4 = apoptotic keratinocyte. **(D)** Histological score 3 (viable tissue), mild, 5 = (single) few sloughed cells within eccrine gland. Scale bars: **(A)** 200 μm, **(B–D)** 100 μm.

Supplementary Figure 3 Demonstration of DNA fragmentation (TUNEL) in epidermal cells of the skin explants over a time course of 76 h (viability experiment with mock-infected explants). **(A)** Negative control slide of the TUNEL assay (tissue section not treated with DNAse); **(B)** Positive control slide of the TUNEL assay (tissue section treated with DNAse); **(C)** Skin explant fixed at −8 h (at the abattoir few minutes after animal slaughter); **(D)** 0 h (biopsy fixed at MOCK infection time); **(E)** 28 h (biopsy fixed 28 h after MOCK infection time); **(F)** 52 h (biopsy fixed 52 h after MOCK infection time); **(G)** 76 h (biopsy fixed 76 h after MOCK infection time). Purple nuclei: live cell (TUNEL negative suggesting there was no DNA fragmentation); Brown nuclei: dead cell (TUNEL positive revealing DNA fragmentation). (Scale bar = 50 μm).
