# Supplementary figures and images for: A Novel 3D Skin Explant Model to Study Anaerobic Bacterial Infection
Source: Front Cell Infect Microbiol. 2017 Sep 14;7:404. doi: 10.3389/fcimb.2017.00404 (PMC5604072; doi:10.3389/fcimb.2017.00404)

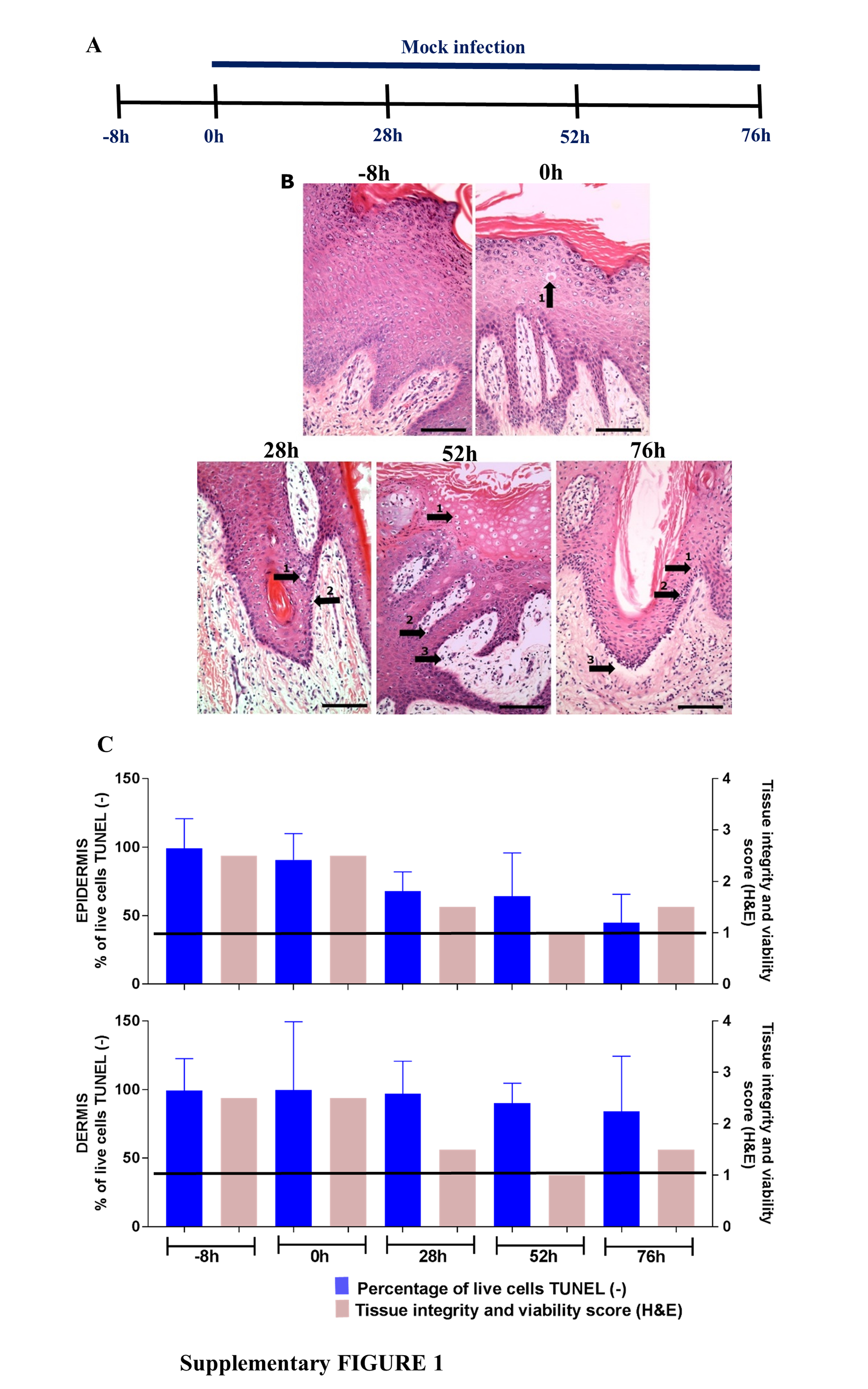

Supplement: Supplementary Figure 1 — Viability of the interdigital skin explants over a time course of 76 h (mock-infected explants). (A) Timeline of viability experiment where one biopsies was placed into RNAlater and one into 10% NBF at each time point. −8h (biopsy fixed at the abattoir a few minutes after sheep slaughter); 0 h (biopsy fixed at MOCK infection time); 28 h (biopsy fixed 28 h after MOCK infection time); 52 h (biopsy fixed 52 h after MOCK infection time); 76 h (biopsy fixed 76 h after MOCK infection time). (B) H&E images illustrating the overall maintenance of the tissue structure and viability over 76 h of incubation. Early signs of tissue degeneration were more marked at 52 and 76 h of incubation including apoptotic keratinocytes (1), basal cell vacuolisation (2), subepidermal clefting (3) (scale bars: 100 μm). (C) Comparison of cell viability (TUNEL) and tissue integrity (H&E scores) in the epidermis and in the dermis of ovine interdigital skin. Histological score 0: tissue is not viable; score 1: viable tissue, but showing marked signs of tissue degeneration; score 1.5: viable tissue, but showing moderate to marked signs of tissue degeneration; score 2: viable tissue, but showing moderate signs of tissue degeneration; score 2.5: viable tissue, but showing mild to moderate signs of tissue degeneration; score 3: tissue is viable and shows only few mild signs of tissue degeneration. Mean and standard deviation of live cells from 5 non-overlapping images from the epidermis are shown. For H&E analysis, 2 slides were analysed for each single biopsy; for all time points, both slides received the same histopathological score. Black horizontal line indicates histopathology score 1, the minimal score for a tissue to be deemed viable. Blue bars show proportion of TUNEL-negative cells. Pink bars indicate tissue integrity and viability score (H&E). Each bar indicates single biopsies. [file Image1.TIF]

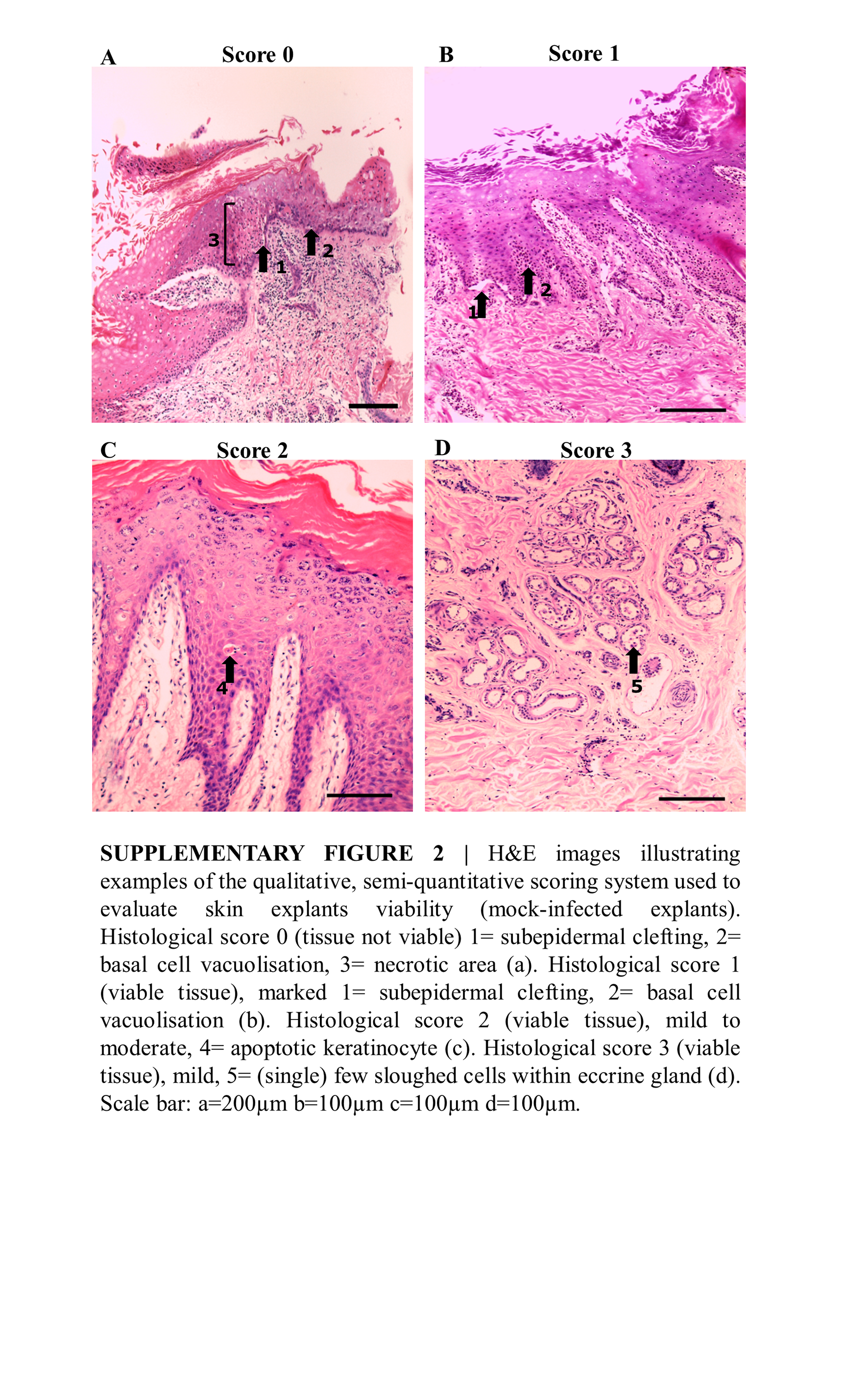

Supplement: Supplementary Figure 2 — H&E images illustrating examples of the qualitative, semi-quantitative scoring system used to evaluate skin explants viability (mock-infected explants). (A) Histological score 0 (tissue not viable) 1 = subepidermal clefting, 2 = basal cell vacuolisation, 3 = necrotic area. (B) Histological score 1 (viable tissue), marked 1 = subepidermal clefting, 2 = basal cell vacuolisation. (C) Histological score 2 (viable tissue), mild to moderate, 4 = apoptotic keratinocyte. (D) Histological score 3 (viable tissue), mild, 5 = (single) few sloughed cells within eccrine gland. Scale bars: (A) 200 μm, (B–D) 100 μm. [file Image2.TIF]

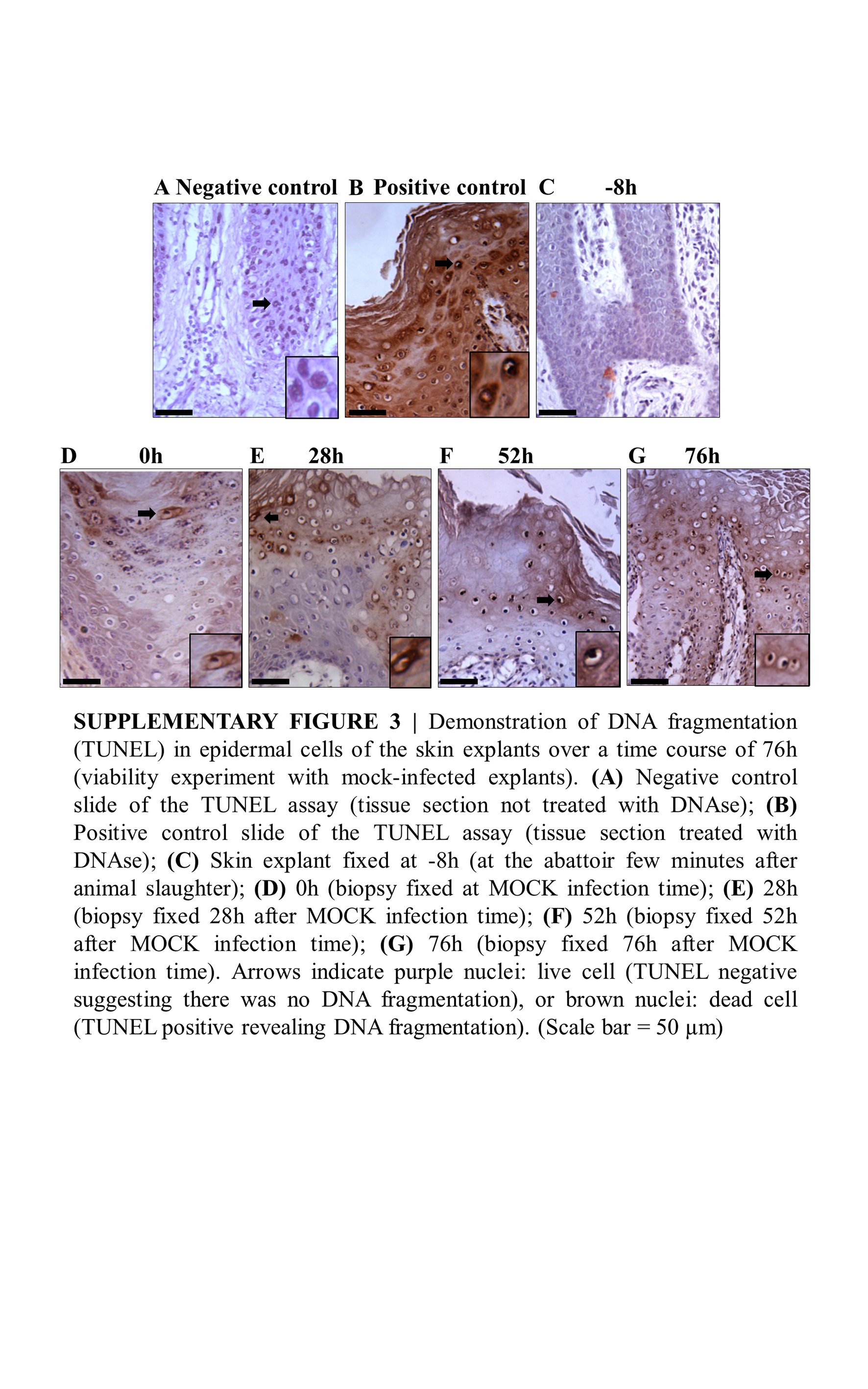

Supplement: Supplementary Figure 3 — Demonstration of DNA fragmentation (TUNEL) in epidermal cells of the skin explants over a time course of 76 h (viability experiment with mock-infected explants). (A) Negative control slide of the TUNEL assay (tissue section not treated with DNAse); (B) Positive control slide of the TUNEL assay (tissue section treated with DNAse); (C) Skin explant fixed at −8 h (at the abattoir few minutes after animal slaughter); (D) 0 h (biopsy fixed at MOCK infection time); (E) 28 h (biopsy fixed 28 h after MOCK infection time); (F) 52 h (biopsy fixed 52 h after MOCK infection time); (G) 76 h (biopsy fixed 76 h after MOCK infection time). Purple nuclei: live cell (TUNEL negative suggesting there was no DNA fragmentation); Brown nuclei: dead cell (TUNEL positive revealing DNA fragmentation). (Scale bar = 50 μm). [file Image3.TIF]
