# Supplementary material for: A Novel 3D Skin Explant Model to Study Anaerobic Bacterial Infection
Source: Front Cell Infect Microbiol. 2017 Sep 14;7:404. doi: 10.3389/fcimb.2017.00404 (PMC5604072; doi:10.3389/fcimb.2017.00404)
Supplement: Supplementary file 4 [file Table1.docx]

**Supplementary Table 1** **|** Haematoxylin and Eosin staining protocol for ovine interdigital skin tissue samples.

| **Reagent** | **Time** |
| --- | --- |
| Xylene | 5 minutes |
| 100% ethanol | 5 minutes |
| 90% ethanol | 5 minutes |
| 70% ethanol | 5 minutes |
| dH_2_O | 5 minutes |
| Haematoxylin | Quick dip |
| Wash under running water |  |
| 1% acid IMS | 2 dips (2-4 seconds each) |
| Wash under running water |  |
| Ammoniated water | 2 dips |
| Wash under running water |  |
| Eosin | 3 minutes |
| Wash under running water |  |
| dH_2_O | 5 minutes |
| 70% ethanol | 5 minutes |
| 90% ethanol | 5 minutes |
| 100% ethanol | 5 minutes |
| Xylene | 5 minutes |
| Mount using DPX |  |
